# Health and care in ageing societies. A new international approach

**Published:** 2012-10-12

**Authors:** Aggie Paulus

**Affiliations:** Department of Health Services Research, Caphri School of Public Health and Primary Care, Maastricht University, Faculty of Health Medicine and Life Sciences, P.O. Box 616, 6200 MD Maastricht, The Netherlands, E-mail: a.paulus@maastrichtuniversity.nl

The ageing of the population results in many debates about viable, effective, efficient and necessary health and social care policies for older people. This book challenges the traditional view that these policies should promote cost containment, functional health and independence in older people by presenting a critical analysis of why it is that the policy agenda has been framed in this manner. Using a feminist ethics framework, the book presents a perspective which is based on the idea that practices of care (including global and local contexts) are closely linked to political and economic agendas. As such, “…it offers an alternative way of conceptualizing health and care…through its explanation of the link between care and health in later life” (p. 139).

The book consists of eight chapters. Besides an introductory and concluding chapter, the first part of the book presents the main concepts (Chapters 2, 3 and 4) while the second part presents several applications (Chapters 5, 6 and 7). In both parts, all chapters end with a summary of the main content.

Chapter 1 describes the main principles of the feminist ethics of care and provides the reasons as to why this framework is relevant for the analysis of policy developments and the interrelationship between ageing, health and care. This chapter also presents an overview of the book.

Using data on life expectancy, mortality and morbidity rates, Chapter 2 describes the patterns in ageing and health. It is argued that a critical approach towards these data is necessary as policies “….both influence and are influenced by the data” (p. 11).

Chapter 3 presents different conceptualizations of health and care and shows the complexity of both concepts. The resulting implications for policy-making are further explored in Chapter 4. In this chapter, the role of policy in shaping the concepts of ageing, health and care is investigated. Policy is presented as a process consisting of “….actions and non-actions, a process of negotiation that creates a shared understanding and perspective on phenomena, such as health and care” (p. 67).

The ways in which these shared understandings apply in the context of health promotion is discussed in the next three chapters. Chapter 5 focuses on the primary level of health promotion and explores a range of factors concerned with the prevention of illness and the promotion of health. Chapter 6 explores the secondary level of health promotion, i.e. a range of treatments and actions to restore health through health care interventions. Chapter 7 describes the tertiary level, i.e. actions that promote health in the context of long-term or incurable illness.

Chapter 8 is the concluding chapter of the book. This chapter identifies the key issues of the book and the value of the analytical framework that was used.

In general as well as for those specifically interested in integrated care for older people, this book is worth reading. The book raises a lot of moral, ethical, political, policy and other questions. As such it challenges traditional views on health and care and presents a new and critical view on policies in ageing societies. Although more attention for applications outside the area of health promotion would have benefitted the book, the book is well structured and an interesting read for policy-makers, social scientists and others involved in (interdisciplinary) debates on appropriate policies for older people. Although the book does not explicitly mention integrated care, it does emphasize the importance of the presence of supportive social relationships as a prerequisite for health and the significance of a multisectoral policy approach. Since integration of health and social care sectors is one of the aims of integrated care, this book offers some interesting starting points for discussions on integrated care policy to the readers of this journal as well.

